# Associations between high-density lipoprotein cholesterol levels and computed tomography-defined low muscle mass in older adults and sex-related differences

**DOI:** 10.3389/fendo.2025.1600431

**Published:** 2025-06-11

**Authors:** Weixiao Zhang, Yongkang Liu, Yiping Zhang, Dingzhe Zhang, Jianhua Wang, Xiao Chen

**Affiliations:** ^1^ Department of Nuclear Medicine, Nanjing BenQ Medical Center, The Affiliated BenQ Hospital of Nanjing Medical University, Nanjing, China; ^2^ Department of Radiology, Affiliated Hospital of Nanjing University of Chinese Medicine, Nanjing, China

**Keywords:** low muscle mass, sarcopenia, muscle area, high-density lipoprotein cholesterol, chest CT

## Abstract

**Background:**

The associations between high-density lipoprotein cholesterol (HDL-C) levels and the risk of sarcopenia are inconclusive. This study aimed to investigate the association between HDL-C levels and chest computed tomography (CT)-defined low muscle mass in older adults and its sex-related differences.

**Methods:**

This prospective study involved 1995 participants aged ≥50 years. The muscle area of the bilateral erector spinae muscles was measured at the T12 level on a single CT image. Linear regression analysis was used to evaluate the effects of related factors on muscle area. Multivariate logistic regression and restricted cubic spline (RCS) analysis were used to analyze the relationships between HDL-C quartile and low muscle mass in all participants and in the male and female subgroups.

**Results:**

An increased HDL-C level was associated with a greater risk of lower muscle area overall (β=-1.91, 95% CI: -2.95 to -0.87) and in male participants (β=-3.16, 95% CI: -4.70– -1.61), whereas no significant difference was found in the female subgroup (*P* > 0.05). A higher continuous HDL-C level was associated with a greater risk of low muscle mass in all participants (odds ratio (OR) =2.28, 95% confidence interval (CI): 1.51–3.45) and in the male subgroup (OR=3.28, 95% CI: 1.84–5.87) after adjustment for confounders, whereas no significant difference was found in the female subgroup (P>0.05). Furthermore, the RCS model showed similar results regarding the relationship between HDL-C levels and the risk of low muscle mass.

**Conclusions:**

Higher HDL-C levels were associated with a significantly greater risk of low muscle mass, particularly in older male adults. HDL-C levels are useful in identifying older individuals who are at risk for low muscle mass.

## Introduction

Sarcopenia is a progressive skeletal muscle disorder characterized by accelerated loss of muscle mass and function (1). Sarcopenia is an age-related process that commonly occurs in older adults and is affected by concurrent risk factors and genetic and lifestyle factors throughout the life course (2). Sarcopenia has been shown to predict negative outcomes, including increased risk of osteoporosis, falls and disability, cardiovascular disorders, diabetes, and even mortality (3). In the context of global aging, sarcopenia is becoming an important public health challenge.

Few studies have reported the association between high-density lipoprotein cholesterol (HDL-C) and the risk of sarcopenia (4, 5). Several studies have demonstrated that high levels of HDL-C increase the risk of sarcopenia (4, 5). However, a study reported that sarcopenic patients had significantly lower HDL-C levels than nonsarcopenic patients did (6). Furthermore, one study indicated that there was no significant difference in HDL-C levels between sarcopenia patients and nonsarcopenia patients (7). Those studies indicated that the association between HDL-C and sarcopenia was inconclusive.

In our country, CT lung cancer screening is recommended for subjects aged 50–80 years (8). Subjects at such ages are also susceptible to sarcopenia. It would be interesting to assess low muscle mass during CT chest scans in a timely manner. Computed tomography (CT) is an effective imaging technique for estimating muscle mass by measuring the skeletal muscle cross-sectional area (SMA) (9). However, few studies have reported that chest CT reveals sarcopenia or low muscle mass (10, 11). A recent study demonstrated that the SMA and SMA/height^2^ at the T12 level were strongly associated with whole-body skeletal muscle mass (BSM), revealing that they could be used as surrogates to diagnose sarcopenia (10). However, to our knowledge, studies assessing the associations between HDL-C and chest CT-defined low muscle mass and whether sex differences exist remain insufficient. Thus, this study aimed to explore the associations between HDL-C levels and chest computed tomography-defined low muscle mass in older adults and its sex-related differences.

## Materials and methods

### Study population

This was a cross-sectional study based on 1995 individuals who received routine chest CT scans for lung cancer screening at the Affiliated Hospital of Nanjing University of Chinese Medicine. All participants were aged ≥ 50 years. Individuals who had a history of malignant tumors, severe liver disease and renal dysfunction, rheumatic disease or thyroid disease, which may affect muscle or body metabolism, were excluded from this study. The study was approved by the Ethics Committee of the Affiliated Hospital of Nanjing University of Chinese Medicine. Written informed consent was waived because of the retrospective design.

### Collection of general and blood test data

Information, including age, sex, alcohol consumption status, smoking status, and body mass index information, was collected from the electronic medical system. The following parameters were measured in blood samples after at least 8 h of fasting: high-density lipoprotein-cholesterol (HDL-C), low-density lipoprotein cholesterol (LDL-C), triglyceride (TG), total cholesterol (TC), aspartate aminotransferase (AST), albumin, creatinine, and blood glucose. All of this information was collected from May 2023 to October 2023.

### Measurement of muscle area on unenhanced chest CT

**Figure 1:**
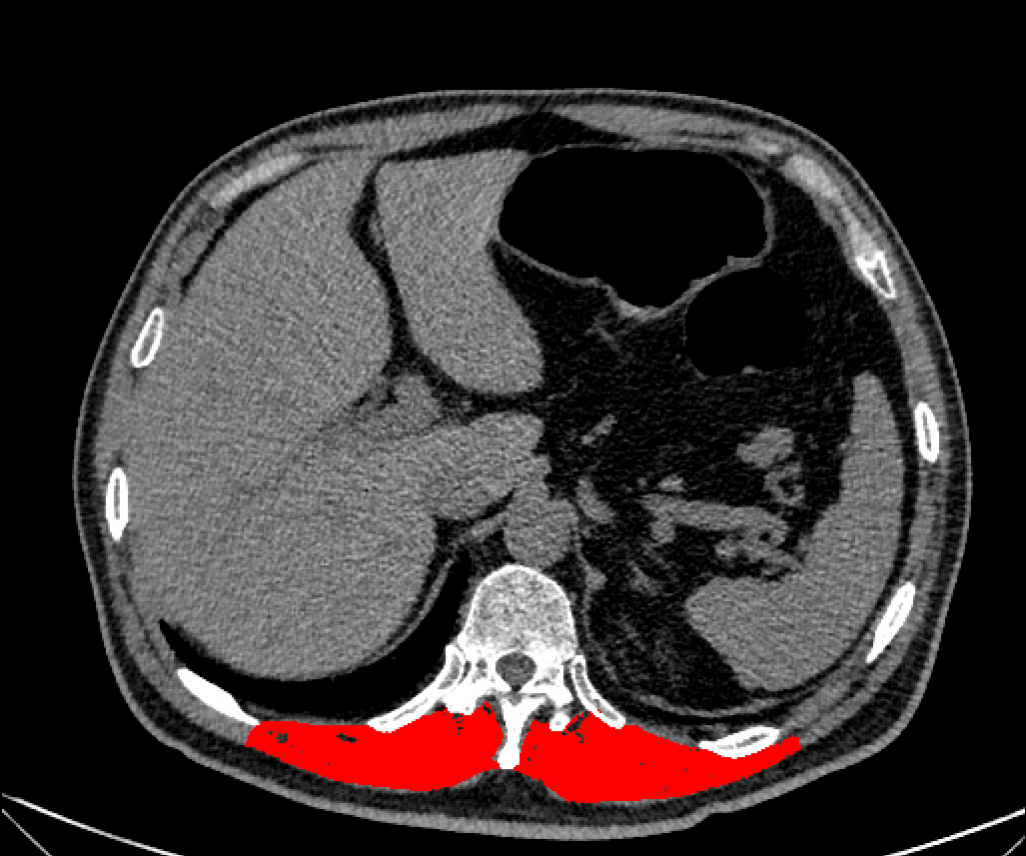
Illustration of the measurement of the muscle area via chest CT.

Unenhanced chest CT was performed for all participants. The CT acquisition parameters were as follows: 120 kV, tube current of 100–120 mAs, slice thickness of 0.625 mm and 512×512 pixels. On a single sagittal CT image at the T12 vertebra level, bilateral erector spinae muscles (iliocostalis, longissimus, and spinalis) were measured via ImageJ software, as shown in **Figure 1**. The muscle tissue thresholds were set at -29–150 (12, 13). Then, ImageJ software automatically segments the muscles within the defined area according to the muscle tissue thresholds, with manual correction of the tissue boundaries when necessary. The 25th percentile has been used as the cutoff point in some studies (14, 15) (Lera et al., 2018; Kidd et al., 2024). Therefore, low muscle mass was defined on the criteria of muscle mass < 25.0 cm² in men and < 20.0 cm^2^ in women (calculated from the 25th percentile of muscle area in men and women). Muscle mass was determined from May 2023 to May 2024.

### Statistical methods

Statistical analyses were performed via SPSS 25.0 software (IBM Corp., Armonk, NY, USA). Continuous and categorical variables are expressed as the means and standard deviations and percentages, respectively. All the subjects were divided into four groups according to HDL-C quartile, and quartile 1 (Q1) served as a reference. One-way analysis of variance or nonparametric rank sum tests were used to compare the levels of related indicators between different groups. Linear regression analysis was used to evaluate the effects of HDL-C on muscle area. Multivariable logistic regression and restricted cubic spline (RCS) analysis were used to analyze the relationships between HDL-C quartile and low muscle mass in all participants and in the male and female subgroups. Three models were analyzed: Model 1 was adjusted for age, sex and body mass index; Model 2 was further adjusted for liver function (AST), renal function (creatinine), diabetes, and albumin. Model 3 was further adjusted for LDL-C, TC and TG. *P* < 0.05 was considered statistically significant.

## Results

### Characteristics of the subjects divided by HDL-C quartile

**Figure 2:**
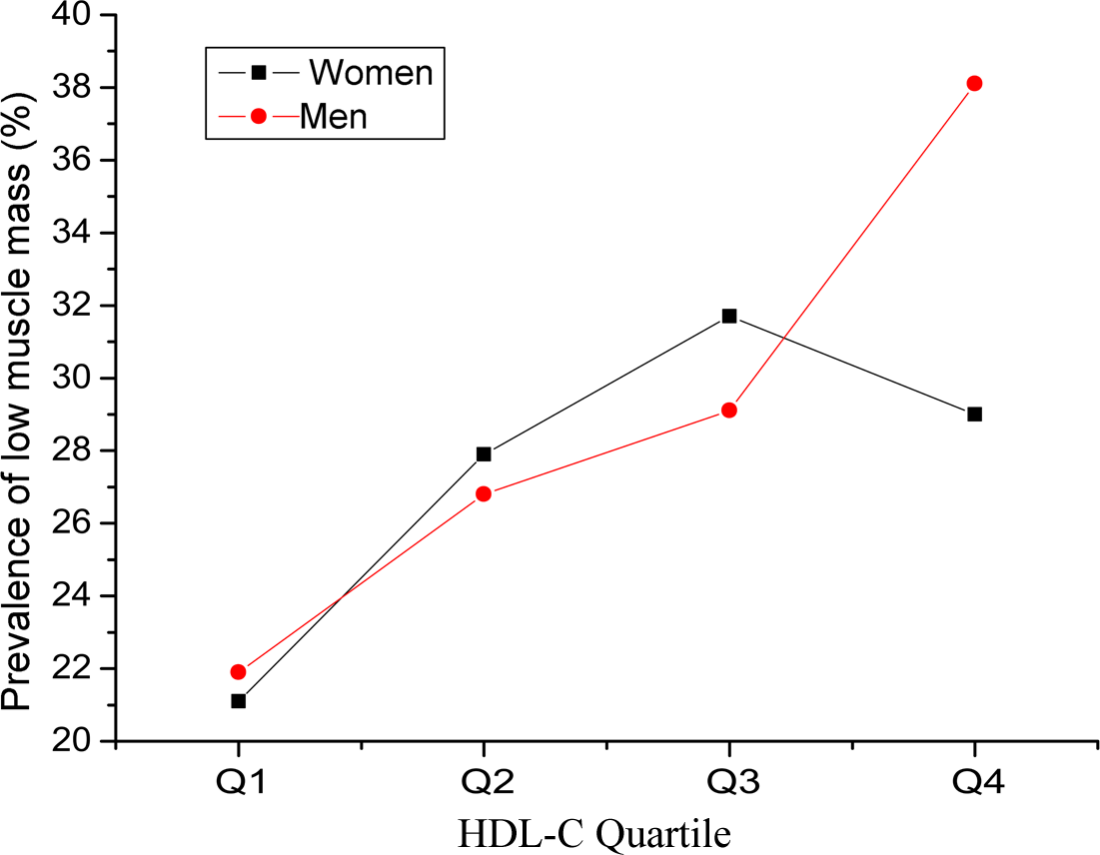
Prevalence of low muscle mass in men and women on the basis of the interquartile range of high-density lipoprotein cholesterol (HDL-C).

The clinical characteristics of the subjects are described in **Table 1**. A total of 1995 subjects were classified into four groups according to the quartile of HDL-C levels. Muscle area, number of individuals with low muscle mass, BMI, creatinine, LDL-C, total cholesterol (TC), total triglyceride (TG), blood glucose, and number of individuals with diabetes were significantly different among the four groups (*P* < 0.05). Compared with those in the Q1 group, the muscle area, BMI, creatinine, TG, blood glucose, and number of diabetic patients in the Q2, Q3 and Q4 groups were significantly lower, whereas the levels of LDL-c, TC, and low muscle mass were significantly greater. The prevalence of low muscle mass in men and women on the basis of the interquartile range of HDL-C is shown in **Figure 2**. The prevalence of low muscle mass increased with increasing HDL-C levels in the male and female subgroups. Compared with individuals in the Q1 group, female participants in the Q2 and Q3 groups with higher HDL-C levels had a greater prevalence of low muscle mass, whereas the prevalence of low muscle mass in the Q4 group was lower than that in the Q3 group. 

**Table 1:** Characteristics of the subjects according to HDL-C quartile.

Variables	Q1 (n = 499)	Q2 (505)	Q3 (n = 501)	Q4 (n = 490)	P
Age (years)	62.83 ± 9.36	61.73 ± 9.11	62.60 ± 9.77	62.41 ± 9.87	0.29
Sex (men)	405 (52.5%)	349 (57.8%)	247 (61.5%)	157 (60.3%)	< 0.001
Muscle area (cm^2^)	29.07 ± 6.94	27.81 ± 6.72	26.12 ± 6.89	24.66 ± 6.27	< 0.001
BMI (kg/m^2^)	27.10 ± 2.92	26.24 ± 2.72	25.53 ± 2.63	24.15 ± 2.06	< 0.001
AST (U/L)	24.10 ± 7.64	23.37 ± 7.10	24.76 ± 7.47	23.92 ± 6.73	0.23
Albumin (g/L)	40.50 ± 3.05	40.71 ± 3.13	40.97 ± 3.18	40.88 ± 3.08	0.09
Creatinine (mmol/L)	83.12 ± 27.92	79.00 ± 15.69	76.01 ± 16.30	72.53 ± 30.31	< 0.001
Blood glucose (mmol/L)	5.70 ± 1.50	5.72 ± 1.69	5.52 ± 1.20	5.26 ± 1.06	< 0.001
HDL-C (mmol/L)	1.10 ± 0.12	1.36 ± 0.07	1.58 ± 0.07	1.98 ± 0.23	< 0.001
LDL-C (mmol/L)	2.71 ± 0.76	2.98 ± 0.81	3.10 ± 0.82	3.01 ± 0.86	< 0.001
TC (mmol/L)	4.26 ± 0.94	4.67 ± 0.90	5.00 ± 0.98	5.24 ± 1.00	< 0.001
TG (mmol/L)	2.04 ± 1.59	1.57 ± 0.83	1.42 ± 0.75	1.11 ± 0.90	< 0.001
Diabetes	56 (11.2%)	54 (10.7%)	37 (7.4%)	17 (3.5%)	< 0.001
CKD	1 (0.2%)	0 (0)	0 (0)	2 (0.4%)	0.29
Low muscle mass	119	129	150	166	0.002

AST, aspartate aminotransferase; BMI, body mass index; CKD: chronic kidney disease; HDL-C: high-density lipoprotein cholesterol; LDL-C, low-density lipoprotein cholesterol; TC, total cholesterol; TG, triglyceride.

### Associations between serum lipids, age, BMI and the risk of lower muscle area

The associations between serum lipid levels, age, BMI and muscle area after adjusting for liver function, renal function, diabetes, and serum ALB are presented in The linear regression results show that an increased HDL-C level was associated with a greater risk of a decreased muscle area overall (β =-1.91, 95% CI: -2.95–0.87) and in male participants (β = -3.16, 95% CI: -4.70– -1.61), whereas no significant difference was found in the female subgroup (β = -0.54, 95% CI: -1.88–0.80, *P* > 0.05) (**Table 2**). Moreover, older age was associated with decreases in muscle area in all participants (β = -0.20, 95% CI: -0.23–0.17), males (β = -0.25, 95% CI: -0.29–0.21) and females (β = -0.13, 95% CI: -0.17–0.09) (*P* < 0.001). No significant correlations were detected between TG or TC levels or BMI and muscle area (*P* > 0.05).

**Table 2:** Relationships between HDL-C and muscle area according to linear regression analysis.

Variables	Total		Women		Men	
	β (95%CI)	p	β (95%CI)	p	β (95%CI)	p
HDL-C (mmol/L)	-1.91 (-2.95 to -0.87)	< 0.001	-0.54 (-1.88 to 0.80)	0.43	-3.16 (-4.70 to -1.61)	< 0.001
TG (mmol/L)	0.21 (-0.08 -0.50)	0.15	0.28 (-0.14 -0.70)	0.20	0.07 (-0.32 -0.47)	0.72
TC (mmol/L)	0.11 (-0.20 - 0.42)	0.50	-0.20 (-0.61 - 0.22)	0.35	0.34 (-0.10 - 0.79)	0.13
Age (years)	-0.20 (-0.23 to -0.17)	<0.001	-0.13 (-0.17 to -0.09)	<0.001	-0.25 (-0.29 to -0.21)	<0.001
Gender	5.58 (4.96-6.20)	<0.001	/	/	/	/
BMI (kg/m^2^)	-0.21 (-0.43-0.06)	0.06	-0.18 (-1.88-0.80)	0.43	-0.12 (-0.39-0.15)	0.39

The model was adjusted for liver function, renal function, diabetes, and serum ALB.

BMI, body mass index; CI, confidence interval; HDL-C, high-density lipoprotein cholesterol; TC, total cholesterol; TG, triglyceride.

### Association between HDL-C level and risk of low muscle mass

The associations between HDL-C levels and the risk of low muscle mass are presented in **Table 3**. A greater continuous HDL-C level was associated with a greater risk of low muscle mass in overall participants (Model 1, OR = 2.01, 95% CI: 1.41–2.89; Model 2, OR = 2.18, 95% CI: 1.50–3.16; Model 3, OR = 2.28, 95% CI: 1.51–3.45) and the male subgroup (Model 1, OR = 2.58, 95% CI: 1.58–4.22; Model 2, OR = 3.02, 95% CI: 1.79–5.09; Model 3, OR = 3.28, 95% CI: 1.84–5.87) after adjustment for different possible confounders, while no significant difference was found in the female subgroup (P>0.05). For female subjects older than 60 years, higher continuous HDL-C levels were associated with a greater risk of low muscle mass in Model 1 (OR = 2.19, 95% CI: 1.07–4.48) and Model 2 (OR = 2.06, 95% CI: 1.00–4.27) but not in Model 3 (OR = 2.01, 95% CI: 1.08–4.70).

**Table 3:** Association between HDL-C and the risk of low muscle mass according to logistic regression analysis.

Quartiles	Model 1	p	Model 2	p	Model 3	p
	OR (95%CI)		OR (95%CI)		OR (95%CI)	
Total						
HDL-C (continuous)	2.01 (1.41-2.89)	<0.001	2.18 (1.50-3.16)	<0.001	2.28 (1.51-3.45)	<0.001
Q1 (<1.25)	1		1		1	
Q2 (1.25-1.47)	1.22 (0.90-1.66)	0.96	1.23 (0.91-1.67)	0.18	1.21 (0.89-1.66)	0.23
Q3 (1.47-1.71)	1.41 (1.03-1.92)	0.033	1.44 (1.05-1.98)	0.023	1.43 (1.03-2.01)	0.035
Q4 (> 1.71)	1.85 (1.31-2.60)	<0.001	1.91 (1.35-2.72)	<0.001	1.92 (1.31-2.82)	0.001
Women						
HDL-C (continuous)	1.63 (0.97-2.74)	0.064	1.62 (0.95-2.75)	0.075	1.55 (0.86-2.79)	0.13
Q1 (<1.43)	1		1		1	
Q2 (1.43-1.64)	1.50 (0.95-2.37)	0.08	1.50 (0.94-2.38)	0.09	1.47 (0.92-2.36)	0.11
Q3 (1.64-1.88)	1.87 (1.18-2.96)	0.008	1.84 (1.16-2.93)	0.01	1.79 (1.09-2.94)	0.02
Q4 (> 1.88)	1.75 (1.06-2.89)	0.028	1.74(1.05-2.89)	0.033	1.73 (1.00-3.00)	0.05
Men						
HDL-C (continuous)	2.58 (1.58-4.22)	< 0.001	3.02 (1.79-5.09)	<0.001	3.28 (1.84-5.87)	<0.001
Q1 (<1.18)	1		1		1	
Q2 (1.18-1.36)	1.23 (0.83-1.84)	0.30	1.23 (0.83-1.84)	0.30	1.21 (0.80-1.82)	0.37
Q3 (1.36-1.56)	1.50 (1.01-2.25)	0.048	1.52 (1.01-2.29)	0.024	1.51(0.98-2.32)	0.06
Q4 (> 1.56)	1.99 (1.30-3.03)	0.001	2.13 (1.38-3.30)	0.001	2.13 (1.33-3.43)	0.002

Model 1 was adjusted for age, sex and body mass index; Model 2 was further adjusted for liver function, renal function, diabetes, and albumin. Model 3 was further adjusted for low-density lipoprotein cholesterol, total cholesterol and triglycerides.

CI, confidence interval; HDL-C, high-density lipoprotein cholesterol; OR, odds ratio.

All the subjects were then divided into four groups according to the HDL-C quartiles, and quartile 1 (Q1) served as a reference. Compared with individuals in the reference Q1 group, overall, participants in the Q3 and Q4 groups with higher HDL-C levels had a greater risk of low muscle mass (OR = 1.43, 95% CI: 1.03–2.01; OR = 1.92, 95% CI: 1.31–2.82). No significant difference was found in the hazard of lower muscle mass between the Q2 group and the reference Q1 group. The subgroup analysis for male and female participants revealed similar results.

**Figure 3:**
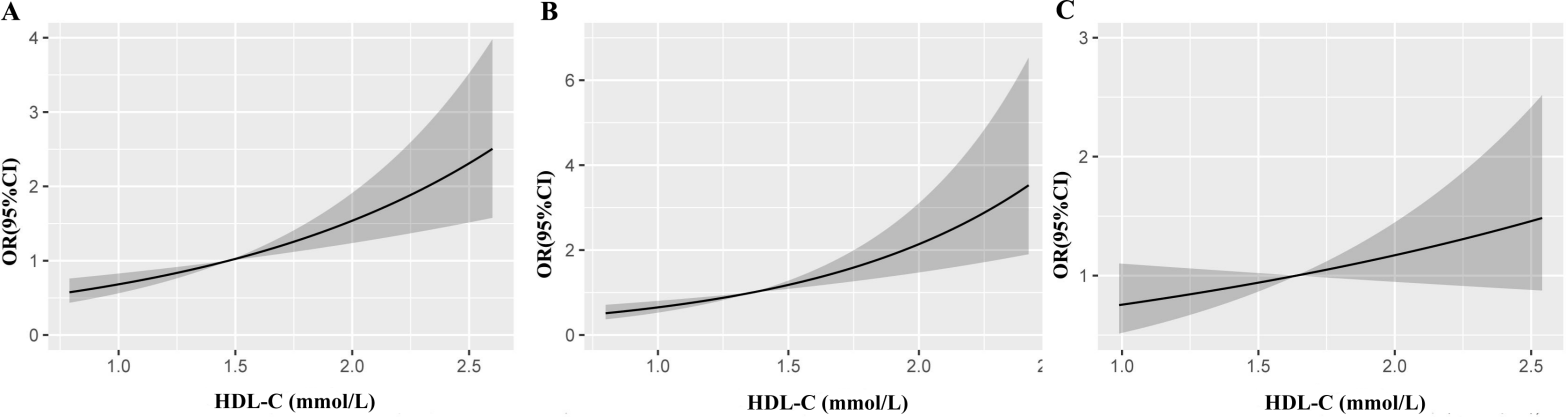
Restricted cubic splines show the multivariable adjusted odds ratio for the risk of low muscle mass according to high-density lipoprotein cholesterol (HDL-C) in the total population **(A)**, men **(B)** and women **(C)**. Age, sex and body mass index, liver function, renal function, diabetes, low-density lipoprotein cholesterol, triglyceride, albumin and total cholesterol were adjusted. CI, confidence interval; OR, odds ratio.

Furthermore, the RCS model revealed a linear relationship between HDL-C and the risk of low muscle mass in all subjects (**Figure 3A**) and in the male subgroup (**Figure 3B**) (*P <*0.001), and these results implied that the risk of low muscle mass increased with increasing HDL-C levels, while this linear relationship was not found in the female subgroup (**Figure 3C**) (P>0.05). The dose–response relationships between HDL-C and the risk of low muscle mass were consistent with the results of the logistic model.

## Discussion

This cross-sectional study of older Chinese adults demonstrated that higher HDL-C levels were associated with a significantly greater risk of low muscle mass, as measured by chest computed tomography. In addition, higher HDL-C levels had a more significant effect on low muscle mass risk in older male adults than in females. Furthermore, the RCS model revealed a linear relationship between HDL-C and the risk of low muscle mass in all subjects and in the male subgroup, whereas this linear relationship was not found in the female subgroup. Our study provides new evidence of the negative effect of very high HDL-C on muscle mass in older adults and suggests that the HDL-C level is an important monitoring index for identifying older individuals who are at risk for low muscle mass.

In this study, higher HDL-C levels were associated with a significantly greater risk of low muscle mass in older Chinese adults, which is consistent with the findings of a subset of extant studies (5, 16, 17). A longitudinal study encompassing 4031 elderly Chinese individuals indicated that older adults with higher HDL-C levels (>70 mg/dl) were at a significantly increased risk of developing sarcopenia and having low grip strength (5). Another 4-year longitudinal study conducted on 7,415 Chinese middle-aged and older adults indicated that, with each 1-unit increase (1SD = 15.4 mg/dL) in HDL-C, the risk of developing sarcopenia increased by 42% at the 4-year follow-up (16). A cross-sectional study including 4302 patients also revealed that sarcopenia patients had lower TG and LDL-C levels but higher HDL-C levels (17).

Nonetheless, findings from certain studies are inconsistent with our findings. For example, data from the National Health and Nutrition Examination Survey (NHANES) involving 4,636 subjects revealed that, in comparison with the control group, the sarcopenia group presented higher LDL-C, TG and total cholesterol (TC) levels and lower HDL-C levels (P <0.05) (18). A probable explanation for the different trends reported in the studies described above is that the impact of HDL-C on sarcopenia is a long-term process, as is the case with cardiovascular disease (19). Although HDL-C is generally acknowledged as the “good cholesterol” beneficial to cardiovascular health, pharmaceutical trials that aim to increase the level of circulating HDL-C have shown a limited capacity to reduce cardiovascular risk (20, 21). Owing to the brevity of cross-sectional studies’ duration, longitudinal associations were insufficiently captured, leading to biased estimates in opposite directions (17, 18). Additional factors contributing to the different outcomes include sample size, the characteristics of the research subjects, and inadequate adjustment for confounding factors. Our findings were consistent with recent longitudinal studies, which reinforced the evidence that high HDL-C levels increase the risk of sarcopenia (5, 16).

Both extremely low and high levels of HDL-C are related to a higher rate of mortality (in terms of total, coronary heart disease and stroke) and an elevated concentration of inflammatory factors (22). This provides an explanation for the differences in the results of the abovementioned observational studies (5, 16–18). A potential interpretation for the association between exceedingly high HDL-C and the risk of low mass is that individuals with exceedingly high HDL-C levels, genetic variants such as CETP (23), scavenger receptor class B member 1 (SCARB1) (24), the hepatic lipase gene (LIPC) (25), and ATP-binding cassette subfamily A member 1 (ABCA1) (26) might be carried. These genetic variants not only increase HDL cholesterol levels but also affect human physiology and thereby potentially increase the risk of disease or death. Mendelian randomization studies may be useful for revealing the causal relationship between high HDL-C and sarcopenia risk. Another possible explanation is that HDL is commonly known for its cardioprotective benefits, since it has anti-inflammatory, antioxidative, antithrombotic, and cytoprotective characteristics (27, 28). Modifications in the HDL lipidome and proteome, including oxidation and glycation, are capable of altering the composition of HDL. HDL subsequently becomes a proinflammatory and atherogenic molecule that is harmful in a variety of pathologies (29–31). Future studies are needed to investigate the effects of high levels of HDL-C on inflammation. Mounting evidence shows that the composition and function of HDL-C are more critical determinants than HDL-C levels for disease outcome, not only in coronary heart disease but also in other conditions (32). The conformation and function of HDL might also be modified in those who have extremely high HDL-C. A hypothesis could be that for those with extremely high HDL-C, the function of HDL-C is undermined so that HDL-C cannot operate properly and instead has detrimental effects (33). Whether there is a causal relationship between extremely high HDL-C levels and increased risk of low muscle mass remains an important unresolved question and demands further in-depth investigation.

We detected heterogeneity in the dose–response association between HDL-C levels and the risk of sarcopenia across different sexes. Our study revealed a linear relationship between HDL-C and the risk of low muscle mass in all subjects and in the male subgroup, whereas this relationship was weak in the female subgroup. The possible mechanisms are unknown. Such sex differences were also observed in some studies (16, 34). A possible explanation for this difference could be the modifying effect of sex hormones on the relationship between HDL-C and sarcopenia (35). With increasing age, the decrease in androgen levels curtails skeletal muscle protein synthesis (36). Similarly, a reduction in estrogen levels may be correlated with an increase in TNF-α, IL-6, and a range of other inflammatory factors, and it can also induce mitochondrial dysfunction, thereby leading to a decrease in muscle mass (37). The decrease in estrogen in women may be greater than the decrease in androgen in men. Estrogen may play a more important role in muscle than does HDL-C in women. Therefore, the linear relationship between HDL-C and sarcopenia in women was diminished. However, as categorical data, the Q2 of HDL-C indicated a high risk of low muscle mass. The relationship between HDL-C may be complicated. Future research is expected to explore the causal mechanisms of this sex-related relationship.

However, there were certain limitations. First, owing to the observational study design, we were unable to establish a causal association between HDL-C levels and muscle mass. We only discuss possible reasons for the association between exceedingly high HDL-C and low muscle mass. To elucidate the pathophysiological underpinnings of these findings, additional prospective or longitudinal studies are imperative. Second, several guidelines have shown that the diagnosis of sarcopenia should be established on the basis of the criteria of low appendicular skeletal muscle mass and compromised muscle function (3). However, owing to insufficient data availability, functional parameters such as handgrip strength were not evaluated in our study. Third, this study focused on the Chinese elderly population. Given the physiological and environmental variances across different ethnic groups, the generalizability of the findings may be limited, and additional research involving multiethnic populations is imperative. Fourth, our population included people who underwent CT lung cancer scans. There were no specific predispositions to lung cancer except for age. To some extent, our population can represent the general population. Although this scan is recommended for all subjects older than 50 years, selection bias cannot be avoided. In addition, we discuss possible mechanisms by which high HDL-C is related to low muscle mass, and further studies are needed to confirm these hypotheses. Furthermore, although the research endeavored to account for potential confounding variables, other variables that could influence the relationship between HDL-C and muscle mass, such as dietary habits and physical exercise, were not considered.

Our research revealed a linear correlation between HDL-C levels and the risk of sarcopenia among older individuals, and higher HDL-C levels were significantly associated with an increased risk of low muscle mass. Increasing HDL-C levels had a more significant effect on low muscle mass risk in older male individuals. HDL-C may be useful in identifying older individuals with sarcopenia or for managing sarcopenia in older adults.

## Data Availability

The raw data supporting the conclusions of this article will be made available by the authors, without undue reservation.
